# Large-area and low-cost SERS substrates based on a gold-coated nanostructured surface fabricated on a wafer-scale†

**DOI:** 10.1039/d2ra00407k

**Published:** 2022-03-28

**Authors:** Abhijit Das, Nitin Gupta, Ajay Kumar Agrawal, Anuj Dhawan

**Affiliations:** Department of Electrical Engineering, Indian Institute of Technology Delhi Hauz Khas New Delhi 110016 India adhawan@ee.iitd.ac.in

## Abstract

This paper demonstrates a method to fabricate plasmonic nanostructures over a large area that can be implemented as SERS substrates. The proposed method comprises batch processes such as spin coating, reactive ion etching, and thin metal deposition. These processes can be performed on large wafers, resulting in large numbers of SERS substrates in a single run. The effects of different process parameters were studied to optimize the performance of the SERS substrates. The study of sensitivity on the optimized SERS substrates was conducted using the SERS-active molecule pMBA. The SERS substrates thus fabricated were able to detect molecule concentrations as low as 100 nM. The SERS substrates were also evaluated for uniformity across the sample and for sample-to-sample reproducibility. Finally, the SERS substrates were applied to demonstrate label-free detection of organophosphorous pesticides – paraoxon ethyl and paraoxon methyl.

## Abstract 2

A simple and novel fabrication process for fabricating a uniform and reproducible SERS substrate over a large area.

## Graphical abstract

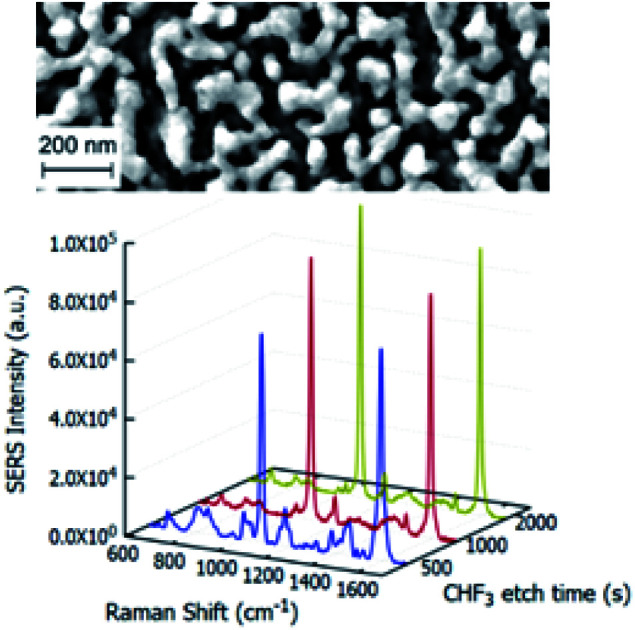

## 1. Introduction

Surface-enhanced Raman spectroscopy (SERS) has emerged as a powerful technique to detect and image biological^1,2^ and chemical species^3,4^ due to its ultrahigh sensitivity down to single-molecule level,^5^ molecular specificity, and label-free sensing capabilities.^6^ To compete with existing analytical techniques, highly reproducible SERS substrates must be fabricated at a relatively low cost. The enhancement of the Raman signals in SERS is based on either electromagnetic (EM) enhancement or chemical enhancement. While the EM SERS enhancement takes place due to the plasmon resonance excitation in metallic thin films or nanostructures — leading an enhancement of EM fields around the metallic nanostructures known as hotspots — chemical enhancement of SERS occurs due to charge transfer between metallic nanostructures and molecules. The density and the intensity of the hotspots are controlled by the size, shape, and morphology of the metallic nanostructures on the substrate.^7–10^

The use of noble metal NPs for numerous bioanalysis and detection applications has been extensively studied and reviewed in the literature.^11^ Synthesized AgNPs have been brushed on paper to demonstrate a low-cost implementation for SERS active chips, albeit with high RSD of 13–14%.^12^ pH sensitive SERS paper chips with bimetallic Au nanorod@Ag@Au NPs have also been reported.^13^ Multiple self-assembled methods of AgNP formation on silicon wafers for point-of-use application were studied.^14^ A later work demonstrates the growth of AgNPs *in situ* on silicon wafers, with RSD going up to 11.3%.^15^ Pyramids formed by the template free isotropic etching of silicon *via* KOH/IPA have been combined with post-deposition annealing of silver to obtain SERS substrates.^16^ A self-assembly method for the arrangement of AuNPs has also been demonstrated, but with widely variable RSD going up to 15.4%.^17^ Work has also been done to increase the reproducibility by using molecular linker cucurbit[*n*]uril: CB[*n*] to bind AuNPs at fixed interparticle spacing in aqueous suspension.^18,19^ A combination of nanodimpled substrate and AuNPs was used to create a MIM-like localized region and thus promote sensitivity for SERS detection.^20^

To produce highly uniform and reproducible substrates, lithography techniques such as electron beam lithography (EBL) and focused ion beam (FIB) milling are employed to create an array of nanostructures with narrow gaps and small features with nm precision.^21–24^ However, despite being capable of fabricating SERS substrates that produce uniform SERS signals over a large area, EBL and FIB milling are not attractive to fabricate SERS substrates due to the low throughput of these techniques, which results in expensive SERS substrates.^25^ To overcome this problem, other methods such as self-assembly of metal nanoparticles,^26^ metal film on self-assembly of nanoparticles,^27,28^ and nanosphere lithography^29,30^ have been explored extensively to fabricate cost-effective SERS substrates. Because these lithography techniques require the self-assembly of nanoparticles, which can only be achieved over a limited spatial area, the uniformity of SERS signal over a large area is compromised on substrates fabricated *via* these methods.^31,32^ Planar substrates with immobilized metal NPs and nanostructures *via* lithography or template methods for use in biosensing, optical fiber sensing and environmental analyses have been studied.^33^

The naturally occurring columnar structure of the razor clam has also been functionalized using silver thin film and AgNPs for use as SERS substrates.^34^ This work also implements an oil–water interface self-assembly method for the AgNPs to improve uniformity and therefore, the RSD from above 30% to 18–19%. In another method, AgNPs formed on silicon substrates *via* solution process was used to carry out metal-assisted chemical etching (MACE) and thus produce silicon nanowires. These SiNWs were subsequently coated with AgNPs synthesized by photonic reduction to produce SERS substrates.^35^ SERS substrates in the form of nanocones have also been demonstrated by the use of large area self-assembly of polystyrene nanospheres over PMMA, followed by CHF_3_ + O_2_ plasma etching and SiO_2_/Au overcoating.^36^ The performance and reliability of commercial SERS substrates are also presented in literature showing greatly varying RSD typically around 20%, but even going up to 98% for pMBA.^37^ Pesticides play a significant role in agriculture for the prevention or mitigation of infestation in crops. However, their continuous use also adversely affects groundwater. Pollution of the drinking and human-use water sources can lead to animal and human health risks. Guidelines have therefore been established, limiting their presence in drinking water. SERS has been used for the detection of various pesticides.^38,39^ The use of organophosphorous pesticides – paraoxon ethyl and paraoxon methyl are even banned in certain regions due to concerns regarding exposure and potential impact on non-target organisms. Hence, specific detection of the presence of pesticides in drinking and human-use water is very important so that animal and human health risks can be avoided.

In this paper, we present large-area and low-cost SERS substrates based on a gold-coated nanostructured surface fabricated on a wafer scale. We present a novel fabrication process to fabricate the SERS substrates on a large scale — reactive ion etching (RIE) is employed for developing a nanostructured polymeric resist surface on a wafer-scale, and this nanostructured surface is over-coated with gold to fabricate the SERS substrates having gold nanostructures. The fabrication process proposed in this paper is simple — both in terms of lower fabrication cost as well as lower fabrication times as compared to lithography techniques such as EBL and FIB. In our proposed process, we carry out the nano-structuration of the polymeric resist in a controllable manner and then over-coat these nanostructures with gold. The SERS substrates described in this paper are highly sensitive and can be fabricated on a wafer-scale (for 3-inch, 6-inch, 8-inch, or even 12-inch wafers). Based on SERS measurements taken at different locations on a single SERS substrate as well as on different SERS substrates, the SERS substrates proposed in this paper were found to be fairly uniform, with good process control (reproducibility). In this paper, we also discuss optimization of the different fabrication process parameters such as exposure times to the CHF_3_ and O_2_ plasmas so as to obtain the highest possible SERS signals from analyte molecules present on these SERS substrates. The optimized SERS substrates were employed for the label-free detection of organophosphorous pesticides – paraoxon ethyl and paraoxon methyl. Therefore, this large-area fabrication process provides an easy and low-cost method to make sensitive substrates for the trace detection of chemicals using SERS.

## 2. Methods

### 2.1 Fabrication of the gold-coated nanostructured film

**Fig. 1:**
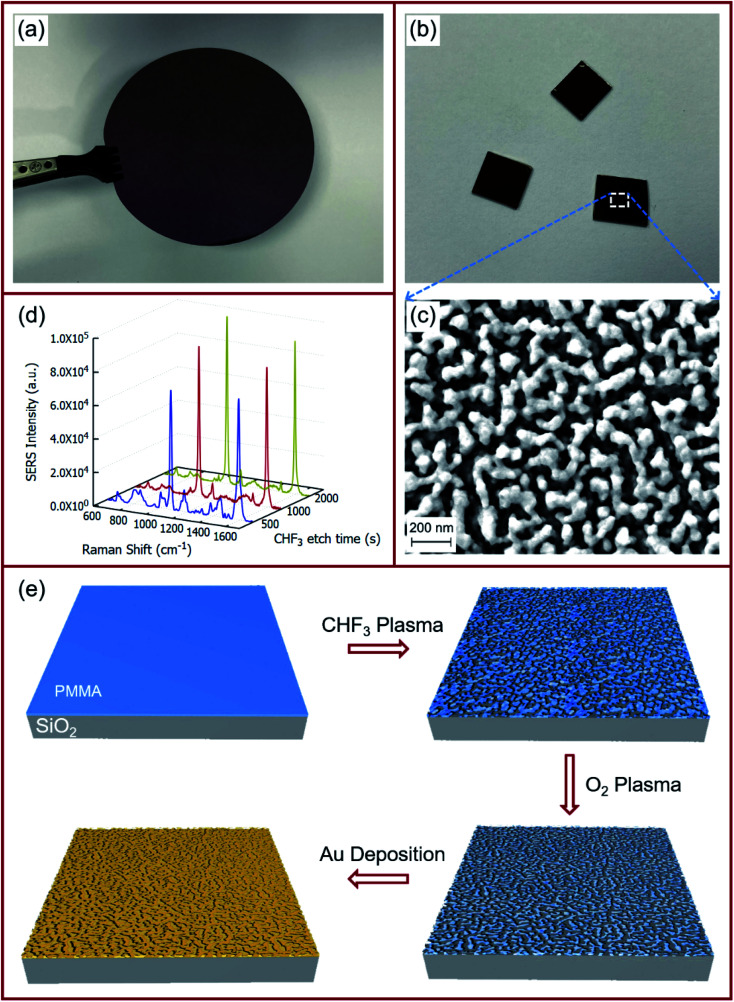
(a) 3′′ silicon wafer with 1000 nm SiO_2_. (b) SERS substrates and (c) SEM images of the gold-coated nanostructured surface formed with 2000 s CHF_3_ plasma exposure, followed by 400 s O_2_ plasma exposure and 60 nm Au overcoating. (d) Raman signals of 10^−3^ M pMBA on the gold-coated nanostructured surface obtained with increasing CHF_3_ plasma exposure followed by 400 s O_2_ plasma exposure and 60 nm Au overcoating. (e) Process schematic for the fabrication of large-area gold-coated nanostructured surface.

A schematic showing the process flow for the fabrication of the gold-coated nanostructured film on SiO_2_ is presented in Fig. 1. A silicon wafer with 1000 nm SiO_2_ thermal oxide was cleaved into pieces of 9 mm × 9 mm. The cleaved pieces were then cleaned in a piranha bath for 15 minutes, followed by rinsing in DI water, drying with high-purity N_2_, and dehydration bake at 180 °C for 10 minutes. PMMA (A4 in anisole, Microchem) was spin-coated at 1500 rpm for 60 s on these cleaned substrates. The thin film of PMMA was baked at 150 °C for 3 minutes to remove any residual solvents. The samples were then placed in an RIE chamber (EtchLab 200, Sentech) and pumped down to a base pressure of 5 × 10^−6^ mbar before further processing. After the base pressure was reached, CHF_3_ was flowed into the chamber at 20 sccm, and chamber pressure was set to 15 mTorr for plasma generation. CHF_3_ plasma was generated in the chamber for an RF power of 100 W. After exposure of the sample for the pre-set period of time, the plasma and the gas were turned off and the chamber pressure allowed to down back down to the base pressure of at least 5 × 10^−6^ mbar before proceeding. Subsequently, O_2_ was flowed into the chamber at 20 sccm, and the chamber pressure was again set to 15 mTorr. O_2_ plasma was generated in the chamber for an RF power of 50 W, and the samples were exposed for another pre-set period of time. Finally, the samples were removed from the RIE chamber and loaded onto another vacuum chamber for thermal evaporation of gold. This chamber was also pumped down to a base pressure of at least 5 × 10^−6^ mbar before proceeding with deposition. Gold (99.999%, ACI Alloys) was evaporated at the rate of 0.1 Å s^−1^ until the desired thickness of 60 nm was reached.

The surface morphology of the samples was determined using field emission scanning electron microscopy (FESEM, Auriga, Zeiss). The samples thus produced were used for further characterization as SERS substrates.

### 2.2 SERS measurements

A confocal Raman microscope (inVia, Renishaw) was used to obtain SERS signals from the substrates. The 785 nm laser was employed to excite the substrates with 12.5 mW power, and data collected for 5 s. SERS signals were collected at room temperature (22 °C) for a range of 600 cm^−1^ to 1700 cm^−1^. The SERS performance of the substrate was studied using 4-mercaptobenzoic acid (pMBA, Spectrochem) as the SERS molecular probe. 10^−3^ M pMBA solution was prepared in ethanol and sequentially diluted for measurements. 10^−4^ M solutions of paraoxon methyl (Analytical Standard, Sigma Aldrich) and paraoxon ethyl (Analytical Standard, Sigma Aldrich) were also prepared in ethanol. The solutions were introduced by drop-casting 20 μL onto the substrate, followed by air-drying before data was collected with 125 mW power for 5 s.

## 3. Results and discussion

### 3.1 Surface morphology

**Fig. 2:**
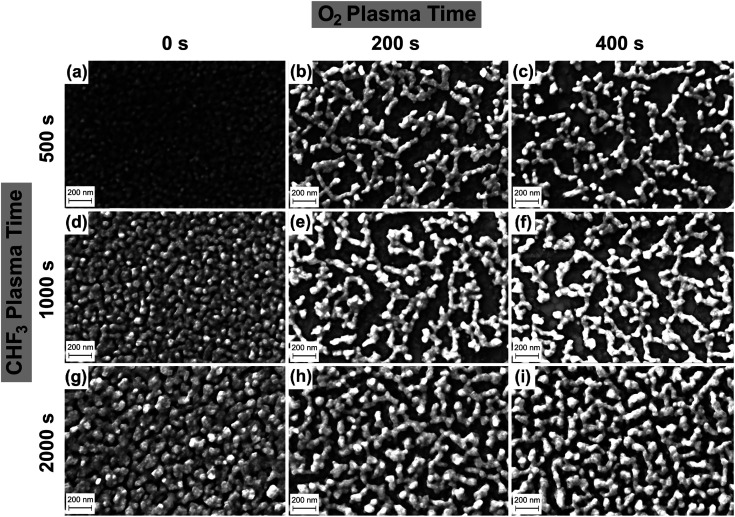
SEM images of the gold-coated nanostructured surface formed with CHF_3_ plasma exposure, followed by O_2_ plasma exposure and 60 nm Au overcoating. When CHF_3_ plasma exposure time is 500 s, the SEM images are shown for different O_2_ plasma exposure times: (a) 0 s, (b) 200 s, and (c) 400 s. When CHF_3_ plasma exposure time is 1000 s, the SEM images are shown for different O_2_ plasma exposure times: (d) 0 s, (e) 200 s, and (f) 400 s. When CHF_3_ plasma exposure time is 2000 s, the SEM images are shown for different O_2_ plasma exposure times: (g) 0 s, (h) 200 s, and (i) 400 s.

FESEM images of the gold-coated nanostructured films on SiO_2_ for varying times of CHF_3_ and O_2_ plasma exposure are presented in Fig. 2. The rows represent increasing times of CHF_3_ plasma exposure, while the columns show increasing times of O_2_ plasma exposure. For instance, Fig. 2(e) shows the morphology of the Au film exposed to the CHF_3_ plasma for 1000 s, followed by O_2_ plasma for 200 s. As observed along the first column, where the samples were only exposed to the CHF_3_ plasma (O_2_ plasma exposure time was NIL), the surface of the Au film becomes increasingly jagged.

The roughness of the spin-coated PMMA film appears to be continuously exacerbated after exposure to the CHF_3_ plasma for longer durations, as some regions are selectively etched more than others. Finally, as the PMMA film becomes thinner from prolonged exposure to the CHF_3_ plasma, the underlying SiO_2_ surface becomes exposed in certain regions. This nanostructured PMMA pattern then transfers onto the SiO_2_ substrate on further plasma exposure. The spin conditions for the PMMA were suitably determined so as to ensure a rough initial surface, to begin with, along with reasonable thickness, so as to ensure uncovering of the SiO_2_ surface underneath within the CHF_3_ etch times. Faster spin speeds lead to thinner but smoother films, while slower speeds lead to rougher and thicker films.

**Fig. 3:**
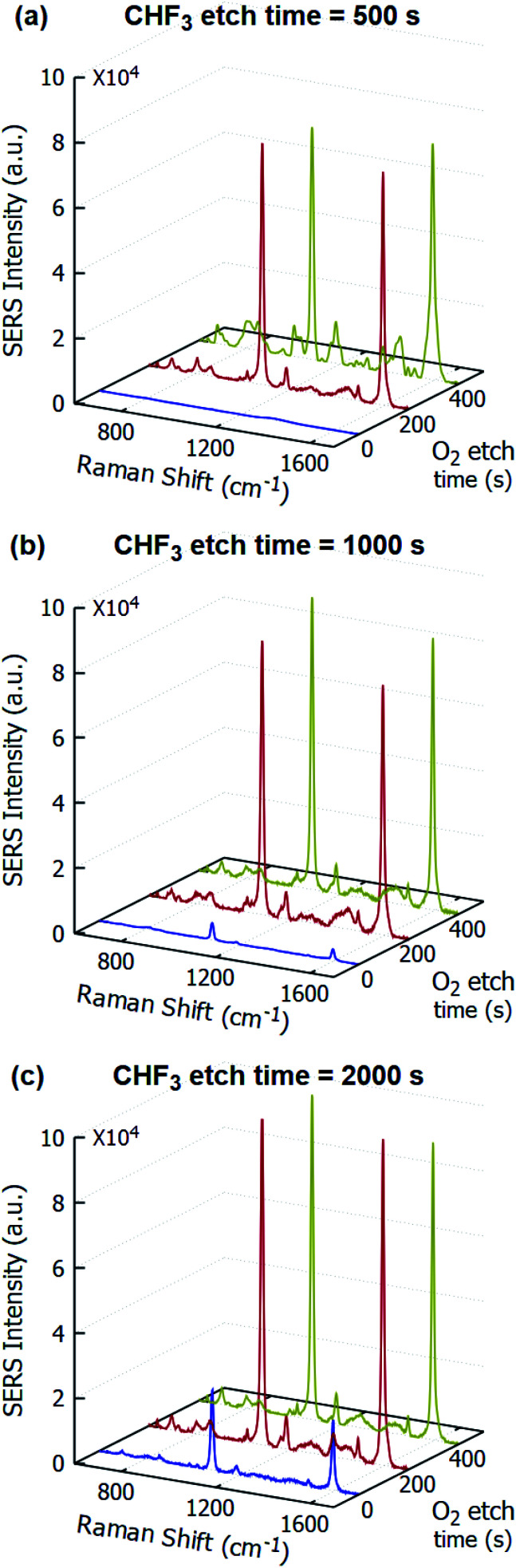
Raman signals of 10^−3^ M pMBA on the gold-coated nanostructured surface obtained with CHF_3_ plasma exposure – (a) 500 s, (b) 1000 s, and (c) 2000 s followed by O_2_ plasma exposure (0 s, 200 s, and 400 s) along *z*-axis.

Subsequently, in the O_2_ plasma, as observed along the rows for increased exposure time, more of the PMMA is removed. The duration of the O_2_ plasma exposure has little to no effect on the samples after CHF_3_ plasma. This can be attributed to the higher etch rates of PMMA, and an almost negligible etch rate of SiO_2_ under O_2_ plasma. This is further verified by the peak intensity of the SERS signals, presented in Fig. 3.

### 3.2 SERS measurements

Fig. 3 shows the SERS signals for 10^−3^ M pMBA on the samples shown in Fig. 2 — each figure for a different CHF_3_ plasma exposure time, showing the variation for the O_2_ plasma exposure time. In both Fig. 3(a) and (c), the intensities of the SERS peaks are almost the same, while in Fig. 3(b), the 200 s exposure time sample has a lower peak intensity than the 400 s sample. This deviation is within the limits of peak intensity variation, as also indicated in Fig. 6 and 7, dealing with location mapping and sample variation. From this, it was concluded that the sample under 2000 s of CHF_3_ plasma exposure and 400 s of O_2_ plasma exposure would be suitable for further study.

**Fig. 4:**
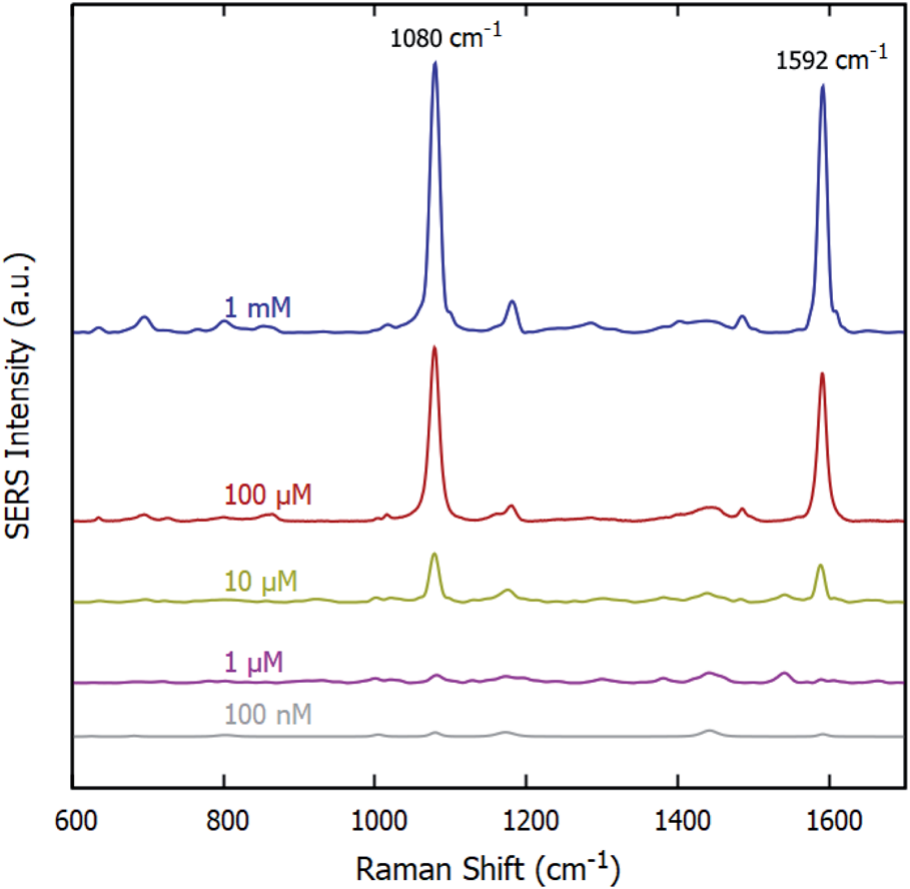
Raman signals of varying concentrations of pMBA on the gold-coated nanostructured surface-based SERS substrates.

**Fig. 5:**
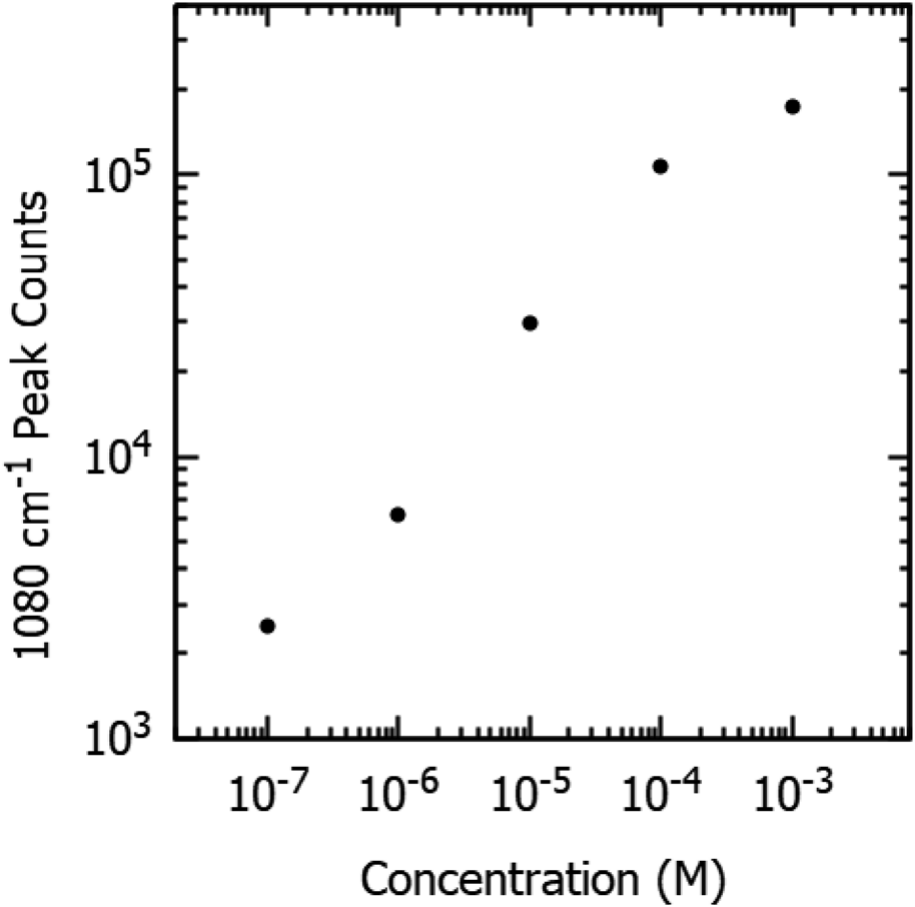
Raman signal peak counts at 1080 cm^−1^ of varying concentrations of pMBA on gold-coated nanostructured surface-based SERS substrates.

The sensitivity of the fabricated SERS substrates has also been examined. Fig. 4 displays the SERS spectra on the optimized SERS substrates for various concentrations of pMBA ranging from 1 mM to 100 nM. The peak intensity of primary bands of pMBA at 1080 cm^−1^ and 1592 cm^−1^ decreases with a decrease in the concentration of pMBA. The peaks at 1080 cm^−1^ and 1592 cm^−1^ are visible for the low concentration of 100 nM. To evaluate the performance of the SERS substrates, we also plotted the intensity of the peak at 1080 cm^−1^ and the concentration on a logarithmic scale, as shown in Fig. 5. The intensity of the Raman peak at 1080 cm^−1^ decreases linearly as the concentration of the pMBA is decreased. The baseline-corrected 1080 cm^−1^ pMBA peak height was used to quantify the performance of the SERS substrates *via* the enhancement factor (EF), defined as:
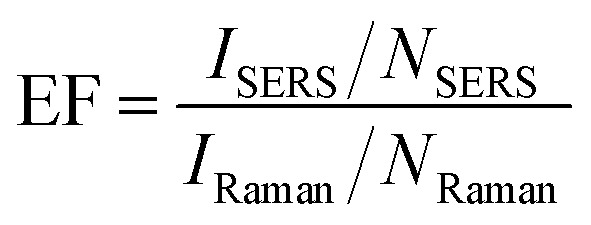
where *I*_Raman_ and *N*_Raman_ are the peak intensity and the number of molecules in aqueous Raman measurements, respectively. *I*_SERS_ and *N*_SERS_ are the peak intensity and number of molecules, respectively, for the SERS measurement. The EF of the gold-coated nanostructured surface-based SERS substrates was evaluated to be 2.11 × 10^5^.

**Fig. 6:**
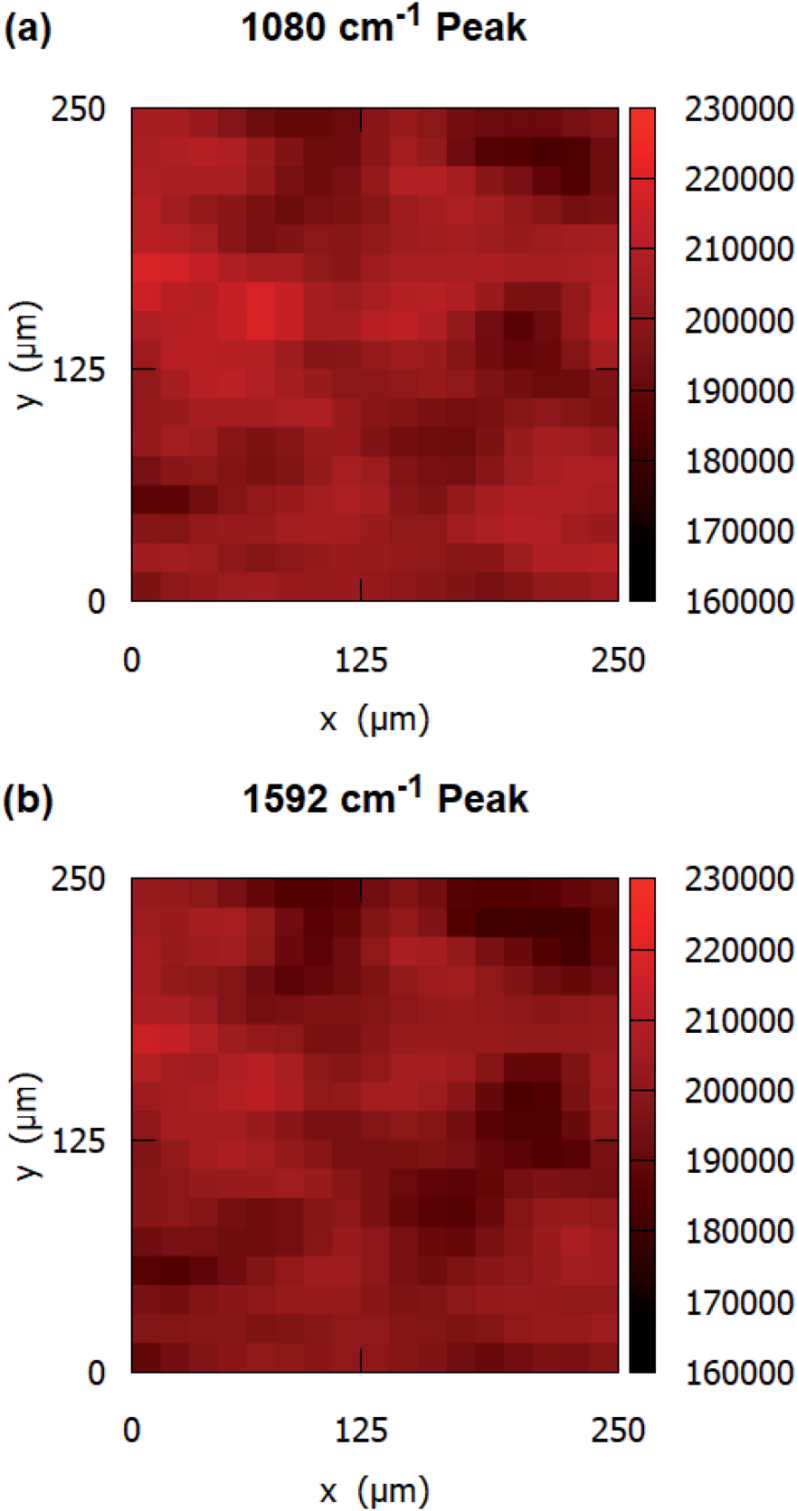
Raman signal peak maps of 10^−3^ M pMBA at (a) 1080 cm^−1^, and (b) 1592 cm^−1^ on gold-coated nanostructured surface-based SERS substrates.

The uniformity and the reproducibility of the SERS substrates were also evaluated. The SERS measurements were conducted at a total of 324 points in an area of 250 μm × 250 μm to obtain Raman signal maps. The intensity variation of two primary peaks of pMBA at 1080 cm^−1^ and 1592 cm^−1^ over an area of 250 μm × 250 μm is shown in the Raman signal maps in Fig. 6(a) and (b), respectively. The SERS substrates show a relative standard deviation (RSD) of 4.32% for 1080 cm^−1^ and RSD of 4.36% for 1592 cm^−1^.

**Fig. 7:**
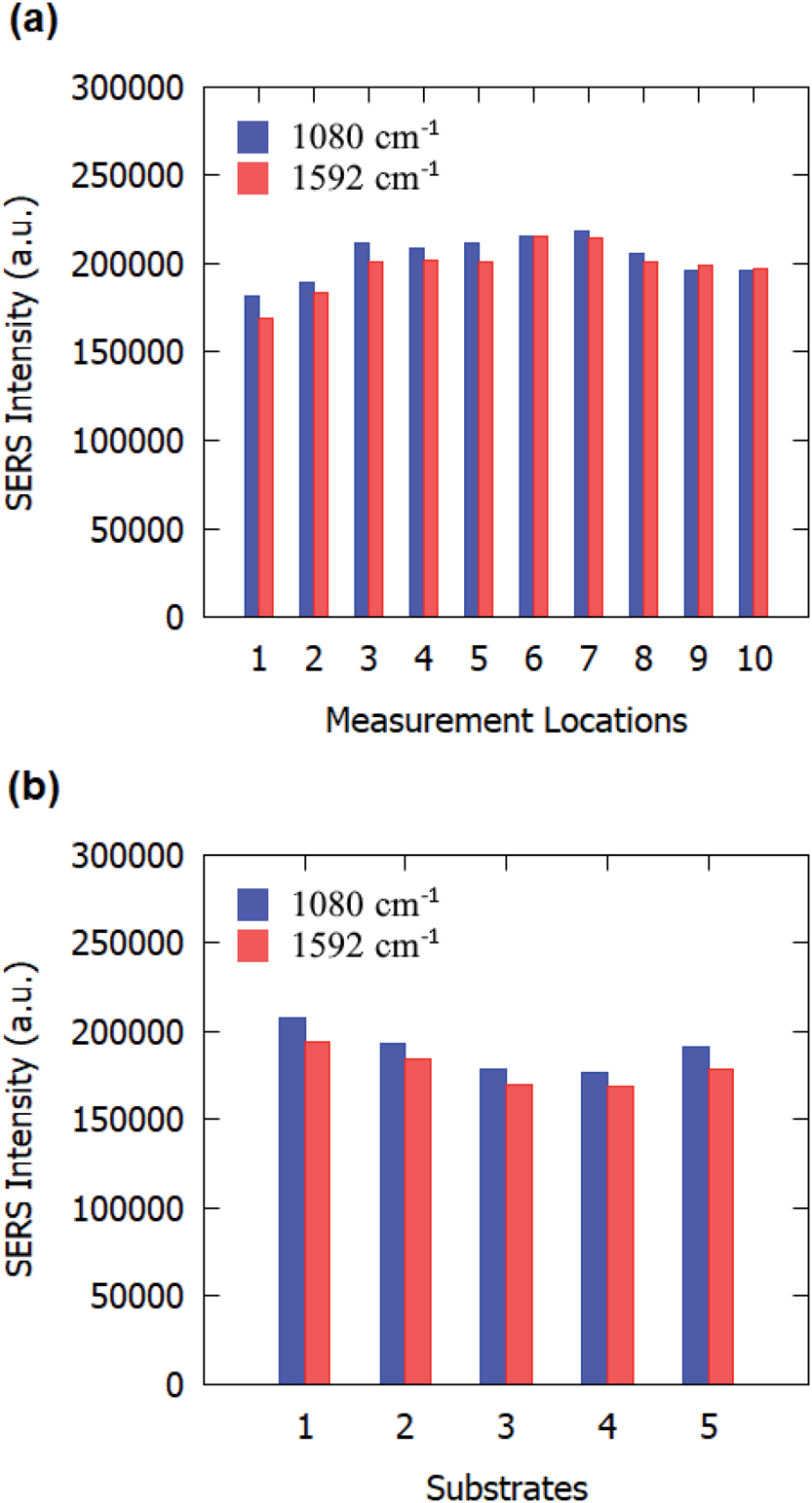
Raman signal peak counts of 10^−3^ M pMBA on gold-coated nanostructured surface-based SERS substrates: (a) at different locations on one sample, and (b) on different substrates.

We also evaluated the uniformity of SERS substrates across the sample area and substrate-to-substrate reproducibility. SERS measurements were conducted at ten random locations on the SERS substrates. The SERS peak intensity at 1080 cm^−1^ and 1592 cm^−1^ at those ten random locations are shown in Fig. 7(a). Calculations reveal that the SERS substrates have an RSD of 5.91% for 1080 cm^−1^ and 6.92% for 1592 cm^−1^ over the ten locations, indicating a good uniformity over the entire substrate. Subsequently, to evaluate the reproducibility of the process, the SERS measurements were taken on five different samples. The SERS intensity for 1080 cm^−1^ and 1592 cm^−1^ peaks of pMBA is plotted in Fig. 7(b) for five different samples. An RSD value of 6.66% for 1080 cm^−1^ and 5.95% for 1592 cm^−1^ were obtained for the five samples, which indicates a good reproducibility of the process. These values of RSD are comparable to previously reported RSD for SERS substrates.^40,41^.

**Fig. 8:**
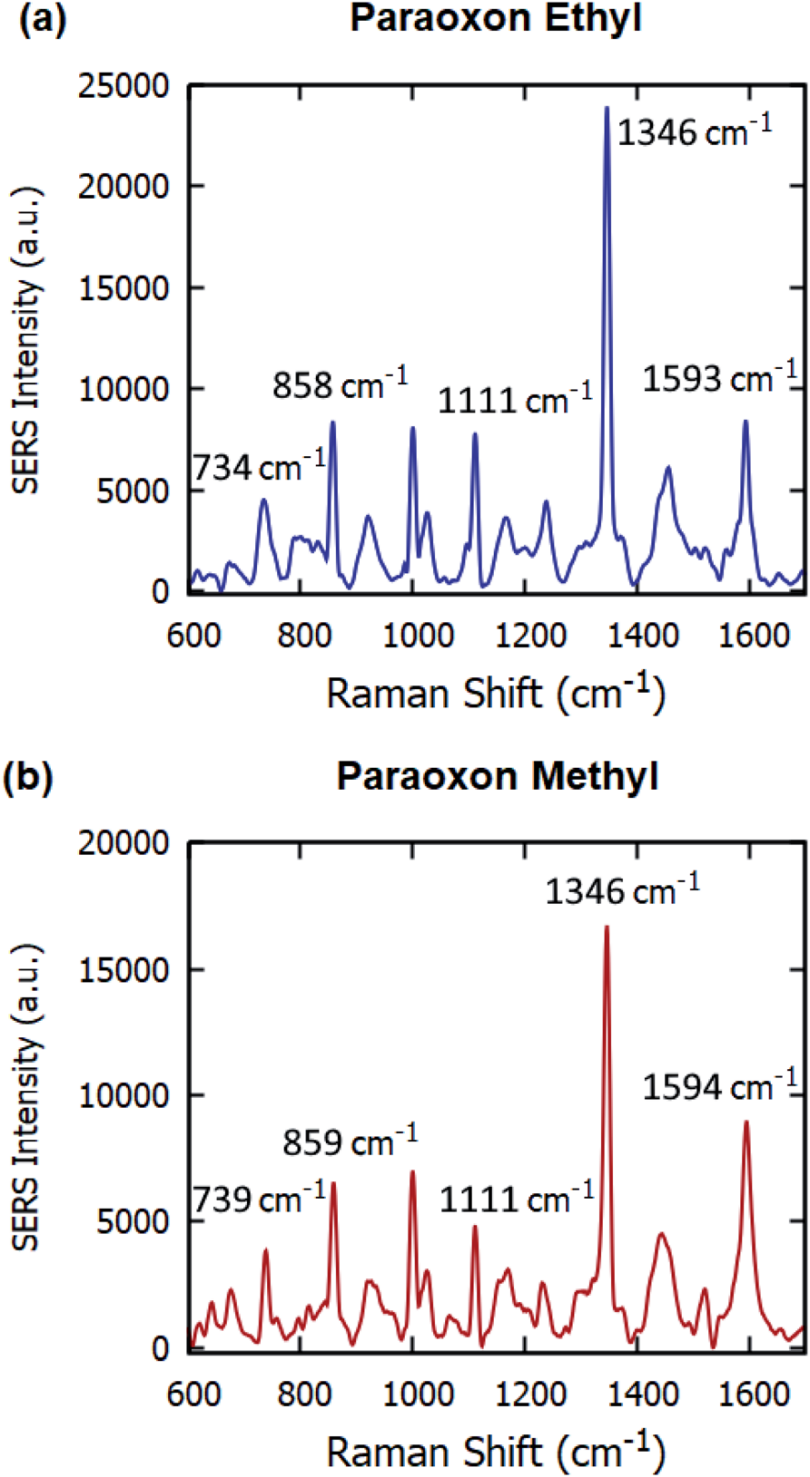
Raman signals of 10^−4^ M (a) paraoxon ethyl and (b) paraoxon methyl on gold-coated nanostructured surface-based SERS substrates.

The SERS substrates developed were used for the label-free detection of organophosphorous pesticides – paraoxon ethyl and paraoxon methyl. 20 μL volume of 10^−4^ M solution was drop-casted on the samples and dried before SERS measurements were taken. The spectrum obtained for paraoxon ethyl in Fig. 8(a) contains multiple peaks – 734 cm^−1^ (NO_2_ scissor and C–C bending vibration), 858 cm^−1^ (NO_2_ scissor vibration), 1111 cm^−1^ (asymmetric NO_2_ stretching vibration, planar C–H vibration), 1346 cm^−1^ (symmetric stretching vibration of NO_2_), and 1593 cm^−1^ (stretching vibration of aromatic ring).^42–44^

For paraoxon methyl in Fig. 8(b), the spectrum has very similar peaks – 859 cm^−1^ (NO_2_ scissor vibration), 1111 cm^−1^ (in-plane C–H bending vibration), 1346 cm^−1^ (NO_2_ symmetric stretching vibration), and 1594 cm^−1^ (aromatic ring vibration).^45,46^ The Raman peak at 739 cm^−1^, attributed to the NO_2_ scissor and C–C bending vibrations, is significantly different than the equivalent peak at 734 cm^−1^ in the spectrum for paraoxon ethyl.

## 4. Conclusions

We present a novel fabrication process for the fabrication of a gold-coated nanostructured surface over a large area which can be employed as SERS substrates. The samples were fabricated by employing a combination of PMMA spin-coating and reactive ion etching, followed by Au evaporation over the etched substrate. The fabrication process proposed in this paper is simple and low-cost — both in terms of lower fabrication cost as well as lower fabrication times as compared to lithography techniques such as EBL and FIB. The process parameters were optimized to maximize SERS signals from the substrate. The optimized SERS substrates were tested using SERS molecular probe pMBA, wherein Raman signals were obtained for concentrations as low as 100 nM. Moreover, the SERS substrates proposed in the paper demonstrate excellent uniformity with RSD of 5.91% for the 1080 cm^−1^ band and 6.92% for the 1592 cm^−1^ band of the pMBA molecule. In addition, the SERS substrates show good substrate-to-substrate reproducibility with RSD of 6.66% for the 1080 cm^−1^ peak of the pMBA molecule. Finally, we also show the detection of organophosphorous pesticides – paraoxon ethyl and paraoxon methyl using the proposed SERS substrates.

## Conflicts of interest

There are no conflicts to declare.

## Supplementary Material

RA-012-D2RA00407K-s001
