# Supplementary material for: Large-area and low-cost SERS substrates based on a gold-coated nanostructured surface fabricated on a wafer-scale
Source: RSC Adv. 2022 Mar 28;12(16):9645–52. doi: 10.1039/d2ra00407k (PMC8959464; doi:10.1039/d2ra00407k)
Supplement: RA-012-D2RA00407K-s001 [file RA-012-D2RA00407K-s001.pdf]

Electronic Supplementary Material (ESI) for RSC Advances.
This journal is © The Royal Society of Chemistry 2022

Supporting Information

Large-area and low-cost SERS sensor chips based on gold-coated nanostructured surface fabricated on a wafer

Details of the Calculation of Enhancement Factor

To estimate the SERS enhancement factor (EF) of pMBA, the characteristic peak at 1080 cm^{-1} was employed in the following equation[1]:

$$EF = \frac{I_{SERS}/N_{SERS}}{I_{Raman}/N_{Raman}}$$

where I_{Raman} and N_{Raman} are the peak intensity and number of molecules in aqueous Raman measurements, respectively. I_{SERS} and N_{SERS} are the peak intensity and number of molecules, respectively, for the SERS measurement.

The parameter N_{Raman} was obtained from the volume illuminated by the laser spot –

$$N_{Raman} = \pi r^2 h(in\ L) \times c_{Raman} \times N_A$$

where, $\pi r^2 h$ is the laser spot volume, c_{Raman} is the solution concentration and N_A is the Avogadro number.
The radius and depth of focus of the laser spot are determined as: $r = 0.61\times\lambda/\mathrm{N.A.}$ and $h = 2\lambda/(\mathrm{N.A.})^2$.[2]
For $\lambda = 785$ nm and N.A. = 0.4, the laser spot volume is 4.416×10^{-14} L.
Therefore, for $c_{Raman} = 1$ mM, we obtain $N_{Raman} = 2.66\times10^7$ molecules.

For the SERS measurement, 20 μL of 10^{-7} M solution was dropped on the substrate, which translates to 2×10^{-12} moles or 1.2×10^{12} molecules of pMBA. These molecules spread over the entire nanostructured substrate. Using AFM measurements and averaged over 4 regions, we evaluated that the ratio of nanostructured area to planar area for the substrate, i.e. the nanostructured area factor, F_A as 1.18. Therefore, the solution spreads over an area of $(9\times9\times1.18)$ mm^2. This yields a pMBA molecular density,

$$\rho_N = \frac{1.2 \times 10^{12}}{9.56 \times 10^{-5}} = 1.26 \times 10^{16}\ molecules/m^2$$

The SERS signals are primarily obtained from the molecules present along the top sidewalls (about 20-30 nm i.e. the limited hotspot area for E-field enhancement) of the nanostructured surface. Therefore, N_{SERS} is obtained as the number of such molecules on the sidewall. The ratio of this sidewall area to planar area of the substrate was evaluated from the nanostructure area factor by assuming a nanostructure depth of 50 nm (typical value obtained from the AFM measurements):

$$sidewall\ factor, F_S = (F_A - 1) \times \frac{20}{50} = 0.072$$

N_{SERS} is therefore calculated as:

$$N_{SERS} = \pi r^2 \times \rho_N \times F_S$$

Using the values obtained for r (radius of the laser spot = $0.61\times\lambda/\mathrm{N.A}$), ρ_N and F_S, we obtain $N_{SERS} = 4.08\times10^3$ molecules

The enhancement factor is therefore evaluated to be –

$$EF = \frac{I_{SERS}/N_{SERS}}{I_{Raman}/N_{Raman}} = \frac{\frac{2495}{12.5\ mW \times 5\ s} \times 2.66 \times 10^7}{\frac{3080}{125\ mW \times 20\ s} \times 4.08 \times 10^3} = 2.11 \times 10^5$$

References

1 E. K. Payne, N. L. Rosi, C. Xue and C. A. Mirkin, *Angew. Chemie - Int. Ed.*, 2005, **44**, 5064–5067.
2 C. A. Tao, Q. An, W. Zhu, H. Yang, W. Li, C. Lin, D. Xu and G. Li, *Chem. Commun.*, 2011, **47**, 9867–9869.
